# Long-Term Results of Laparoscopic Sleeve Gastrectomy: a Review of Studies Reporting 10+ Years Outcomes

**DOI:** 10.1007/s11695-023-06824-8

**Published:** 2023-09-25

**Authors:** Antonio Vitiello, Adam Abu-Abeid, Danit Dayan, Giovanna Berardi, Mario Musella

**Affiliations:** 1 Advanced Biomedical Sciences Department, Naples “Federico II” University, AOU “Federico II” – via S. Pansini 5, 80131 Napoli, Italy; 2 Division of General Surgery, Tel Aviv Sourasky Medical Center, 6 Weizman Street, 64230906 Tel Aviv-Yafo, Israel; 3 Sackler Faculty of Medicine, Tel Aviv University, Tel Aviv-Yafo, Israel

**Keywords:** Sleeve gastrectomy, GERD, Long-term results, Barrett’s disease

## Abstract

Laparoscopic sleeve gastrectomy (LSG) is the most commonly performed bariatric procedure worldwide. Systematic search of Pubmed, Cochrane, and Embase was performed in order to find all the articles reporting 10+ years of LSG results. Eleven studies including 1020 patients met the inclusion criteria. Overall weighted mean %TWL was 24.4% (17–36.9%), and remission rates from TD2M to HTN were 45.6% (0–94.7%) and 41.4% (0–78.4%), respectively. De novo GERD had an overall prevalence of 32.3% (21.4–58.4%), and five cases (0.5%) of Barrett’s disease were reported. Revisional surgery was required for 19.2% (1–49.5%) of patients, Roux-en-Y gastric bypass being the most common secondary procedure.

## Introduction

Obesity pandemic continues to spread worldwide with an estimated prevalence of 51% by 2030 [1]. Metabolic and bariatric surgery (MBS) is the most effective treatment for severe obesity and associated medical problems [2, 3].

Laparoscopic sleeve gastrectomy (LSG) is the most commonly performed MBS since 2014 [4, 5]. It was first introduced by Marceau et al. [6] in 1998 as a modification of the first stage distal gastrectomy of the biliopancreatic diversion surgery. Shortly thereafter in 1999, the laparoscopic approach was applied to sleeve gastrectomy by Ren et al [7]. Considering the accumulating data on its safety and short-term effectiveness, Gumbs et al. [8] recommended in 2007 to perform SG as a stand-alone MBS.

However, LSG has been recently questioned by several studies, whose results have shown a worrisome rate of postoperative gastro-esophageal reflux disease (GERD) [9, 10]. Some articles have also described intestinal metaplasia (Barrett’s disease) after LSG due to the chronic exposure of the lower esophagus to reflux [11]. Evidence of worse outcomes in high BMI patients has also been published [12].

The purpose of this study was to review metabolic outcomes, rates of de novo GERD, and revisional surgery at 10+ years after LSG.

## Methods

### Literature Search

In Pubmed, Embase, and Cochrane Library, a systematic search was performed using the terms “long-term” and “sleeve gastrectomy.” Only original articles in the English language including results at 10 or more years after LSG were included. No temporal interval was set. PRISMA flowchart for reporting meta-analysis [13] was used. References of the articles were further searched to find additional studies. Two independent reviewers performed the screening of titles and abstracts.

### Data Extraction

Following data were extracted using a standardized form: first author and year of publication, study design, type of bariatric surgery, percentage of total weight loss at 10 years (%TWL), remission rates from diabetes (TD2M) and hypertension (HTN), prevalence of de novo gastro-esophageal reflux (GERD) and Barrett’s disease, and percentage and type of revisional surgery.

### Data Analysis

Extracted continuous and categorical variables were reported as mean ± deviation standard and percentages, respectively. Overall means and percentages were calculated weighting the extracted data for the sample size of the article.

## Results

**Fig. 1:**
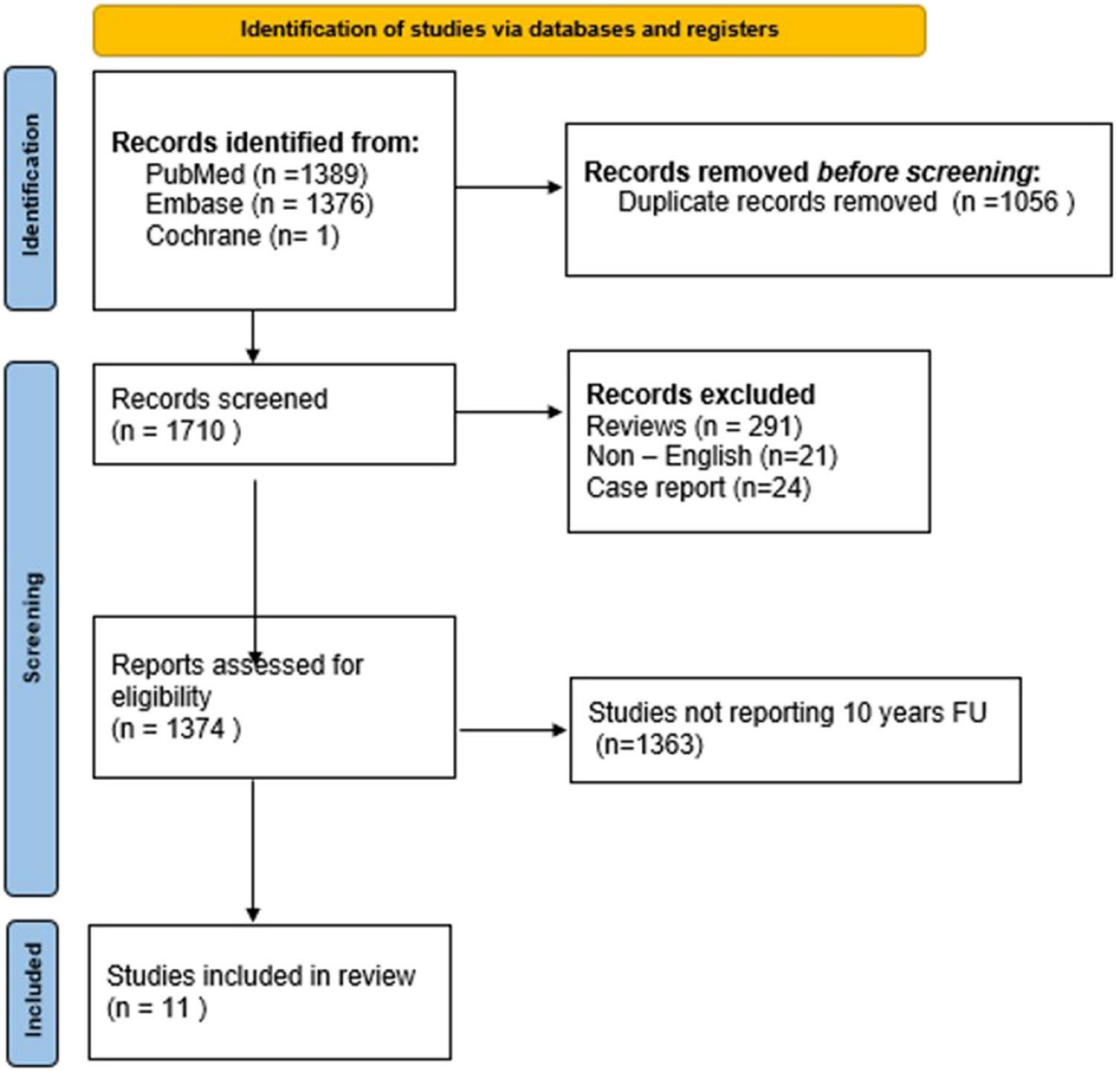
PRISMA flow-chart

The literature search found a total of 2766 articles; 1056 duplicates were removed before screening. After removal of case reports, reviews/meta-analysis and non-English studies 1710 articles on LSG were screened. The PRISMA flow chart for the study selection is shown in Fig. 1. Ten retrospective, [14–23] papers and one randomized controlled study [24] reporting outcomes of LSG after 10 or more years were eventually included in the present study.

Sample size of these articles ranged from 34 to 215 (total number 1020) patients. Only three papers reported a multicenter experience, while the other eight published a single-center case series (Table 1). All articles followed international guidelines on BMI threshold for metabolic surgery [25], and remission of diabetes was considered as fasting blood glucose < 126 mg/dl on two different occasions and as a value of glycated hemoglobin A1c (HbA1C) < 6.55% without necessity for antidiabetic medications [26]. Hypertension remission was defined as blood pressure < 140/90 with no requirement for antihypertensive medication [27]. Follow-up ranged from 44 to 100%, while GERD was diagnosed on the base of symptoms.

**Table 1:** Characteristics of the included studies-, weight loss-, and obesity-related diseases resolution

Author, year	Number of patients	Study design	B.M.I (mean ± SD)	%TWL (mean ± SD)	T2DM resolution	HTN resolution	De novo GERD
Arman, 2016	65	Retrospective multi-center	38.8 ± 7.5	21 ± 12.8	N/A	28.6%*	21.4%
Castagneto Gissey, 2018	114	Retrospective single-center	46.6±7.3	30.9 ± 12.4	64.7%	44.2%	42.9%
Chang, 2018	65	Retrospective single-center	37.9 ± 7.7	26.6 ± 10	39.6%	78.4%	58.4%
Jimenez, 2020	123	Retrospective single-center	46.3 ± 5.1	25.3±11.2	57.7%	14%	N/A
Felsenreich, 2021	53	Retrospective multi-center	48.7 ± 9.2	36.9 ±11.7	0%	50%	18.9%
Hauters, 2021	34	Retrospective single-center	36 ± 8	17 ± 15	12%	17%	41%
Musella, 2021	76	Retrospective single-center	45.1 ± 4.8	22.2 ± 13	0%	51.4%	25.7%
Kraljević, 2021	215	Retrospective single-center	46.4 ± 8.0	21.6 ± 14.1	61%	60.5%	32.4%
Kehagias, 2022	104	Retrospective single-center	43.4 ± 2.9	29 ± 11	94.7%	40%	43%
Avidan, 2023	80	Retrospective single-center	43.86 ± 6.36	19.3 ± 16.7	47%	43.7%	40%
Salminen, 2022	91	Randomized controlled trial, multicenter	47.3	23.4	26%	8%	43%**

*FU* follow-up, *T2DM* type 2 diabetes, *HTN* hypertension, *EWL* excess weight loss, *GERD* gastroesophageal reflux disease, *N/A* not available

*Includes patients with resolution and/or improvement of T2D/HTN

**Includes patients with worsening of preoperative symptoms

Eight (80%) studies reported a %TWL >20 [28] and the overall weighted mean TWL was 24.4% (17–36.9%). Weighted remission rates from TD2M and HTN were 45.6% (0–94.7%) and 41.4% (14–78.4%), respectively. De novo GERD had an overall weighted prevalence of 32.3% (21.4–58.4%) with five cases of Barrett’s disease reported in the eleven included studies (incidence = 0.5%). Revisional surgery was necessary for 19.2% (1–49.5%) of patients and the type of revision was clearly reported in 183 cases. Roux-en-Y gastric bypass (*n* = 123, 67.2%) was the most common secondary procedure, followed by Duodenal Switch (DS; *n* = 36, 19.7%), one anastomosis gastric bypass (OAGB; *n* = 12, 6.6%), single anastomosis duodeno-ileal bypass (SADI-S; *n* = 10, 5.5%), hiatal hernia repair (*n* = 1, 0.5%), re-sleeve (*n* = 1, 0.5%), and banding (*n* = 2, 1%). RYGB and hiatal hernia repair were performed mostly to treat GERD, while OAGB, DS, SADI-S, re-sleeve, and banding were chosen in case of weight persistence/recurrence Table 2.

**Table 2:** Rate and cause of revisional surgery

Author, year	Revisional surgery for GERD	Type of revision for GERD	Revisional surgery for IWL/WR	Type of revision for IWL/WR	Revisional surgery during FU
Arman, 2016	4.8%	RYGB, hiatoplasty	26.9%	RYGB, DS	31.7%
Castagneto Gissey, 2018	1.8%	RYGB	0%	/	1.8%
Chang, 2018	N/A	RYGB, hiatoplasty	N/A	RYGB	21.5%
Jimenez, 2020	N/A	N/A	N/A	N/A	23.8%
Felsenreich, 2021	18.9%	RYGB	26.4%	DS	49.1%*
Hauters, 2021	0%	/	18%	RYGB	18%
Musella, 2021	2.6%	RYGB	13.2%	OAGB	15.8%
Kraljević, 2021	3.9%	RYGB	7.8%	DS	19.2%**
Kehagias, 2022	1%	RYGB	0%	/	1%
Avidan, 2023	0%	/	21.25%	RYGB, OAGB	21.3%
Salminen, 2022	21.1%	RYGB	7.8%	SADI-S	28.9%

*GERD* gastroesophageal reflux disease, *IWL/WR* insufficient weight loss/weight regain, *RYGB* Roux-en-Y gastric bypass, *DS* duodenal switch, *OAGB* one anastomosis gastric bypass

*3.8% were converted due to acute leak

**7.5% were converted due to both GERD and IWL/WR

## Discussion

Despite its growing success, there is an ongoing debate on long-term results of LSG. Specifically, there has always been some skepticism regarding durable effectiveness of restrictive surgery [29] and risks of Barrett’s disease due to de novo GERD [30].

In a previous systematic review published in 2017 on results of SG, Juodeikis and Brimas [31] found that the mean excess weight loss was 58% at 5 years, 78% of patients had resolution of type 2 diabetes, and 68% had resolution of hypertension. In another meta-analysis on the 7 years or more outcomes of SG published in 2018, Clapp et al. [32] found nine relevant studies. The estimated weight regain was 28%, and the estimated overall revision rate was 20%, being weight regain the main cause for reintervention. Surprisingly, in a systematic review and meta-analysis published in 2019 by O’Brien et al. [33] discussing MBS outcomes at 10 years or more, there were only two relevant studies on SG.

The SLEEVEPASS [24] randomized controlled trial demonstrated that although gastric bypass compared to sleeve gastrectomy was associated with greater percentage of excess weight loss at 10 years, rates of BE were comparable.

The current meta-analysis demonstrates that LSG induces outstanding metabolic results at 10+years with %TWL > 20% and satisfactory average remission rates of TDM2 and HTN years. However, two studies reported 0% remission from TDM2 suggesting that LSG could be less effective than other metabolic interventions for the treatment of this disease.

Despite the IFSO task force [34] stated that incidence of Barrett’s esophagus after SG is 4.6% within 5 years, we found only five cases reported out of 1020 patients. However, it is important to note that de novo GERD developed in one third of cases.

Revisional surgery due to weight persistence/recurrence of GERD confirmed to be a significant issue in long-term after LSG. One out of five patients was converted to a secondary intervention within 10 years, being the bypass procedures the most frequent choice as revision.

### Strength and Limitations

Ten out of eleven studies included in this systematic review were retrospective. Follow-up duration varies from 10 to 15 years. There is not a one standard surgical technique for SG which can interfere with outcomes. There are several definitions used for weight regain, which can also affect decision of revision. Not all patients underwent an endoscopy, and it is therefore not possible to determine the precise incidence of BE. Some of the patients included in the abovementioned articles had secondary and not primary SG.

## Conclusion

LSG shows satisfactory results at 10 years in terms of weight loss (%TWL > 20) and remission from HTN. Long-term outcomes of the sleeve gastrectomy on TD2M should be further investigated by prospective trials. Even if one third of patients may develop new onset GERD, less than 20% of individuals requires revisional surgery within 10 years from LSG.
